# Giant Lattice Expansion through Structural Frustration Release in a Dense Oxide

**DOI:** 10.1021/jacs.6c07579

**Published:** 2026-05-20

**Authors:** Zhijun Li, Hongbo Yuan, Alexei A. Belik, Terumasa Tadano, Yoshihiro Tsujimoto, Kazunari Yamaura

**Affiliations:** † Research Center for Materials Nanoarchitectonics (MANA), 52747 National Institute for Materials Science, 1-1 Namiki, Tsukuba, Ibaraki 305-0044, Japan; ‡ Graduate School of Chemical Sciences and Engineering, Hokkaido University, North 10 West 8, Kita-ku, Sapporo, Hokkaido 060-0810, Japan; § Research Center for Magnetic and Spintronic Materials (CMSM), National Institute for Materials Science, 1-2-1 Sengen, Tsukuba, Ibaraki 305-0047, Japan

## Abstract

Large lattice responses in dense inorganic oxides are typically driven by structural phase transitions or redox processes that alter the crystal symmetry or composition. Here, we identify a distinct mechanism for such responses: giant lattice expansion through symmetry-preserving cation redistribution in a metastable, structurally frustrated, dense oxide. High-pressure-quenched Ba_4_Ru_3_O_12_ undergoes an irreversible volumetric expansion of 4.4% between 450 and 650 K while retaining *R*3̅*m* symmetry and oxygen stoichiometry. High-resolution synchrotron diffraction reveals cooperative redistribution of Ru within face-sharing RuO_6_ trimers, directly linking the intratrimer cation configuration to the lattice volume. Thermogravimetric, transport, and magnetic measurements exclude decomposition, redox processes, and electronic or magnetic phase transitions. First-principles calculations show that compression stabilizes a low-volume cation configuration, which is retained after recovery to ambient pressure and relaxes upon heating through intratrimer cation exchange. Together, these results establish symmetry-preserving cation redistribution as a mechanism for giant lattice responses in dense oxides and identify metastable frustrated configurations as a route to large structural responses without symmetry-breaking or compositional change.

## Graphical abstract

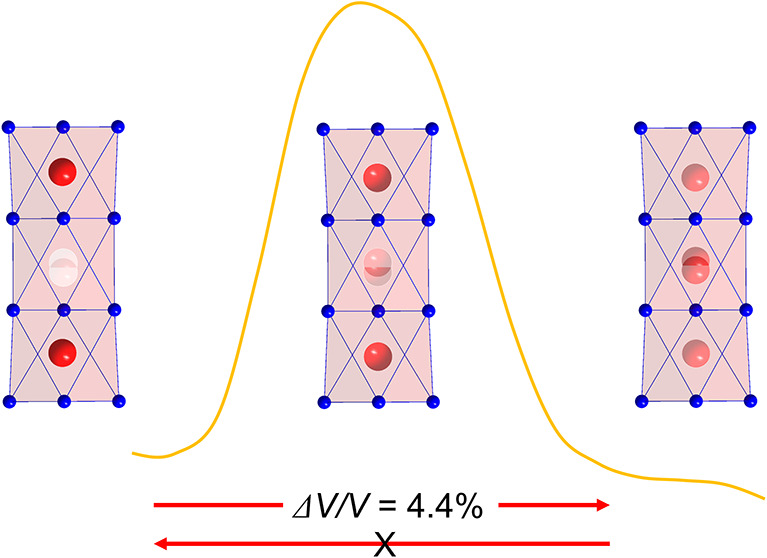

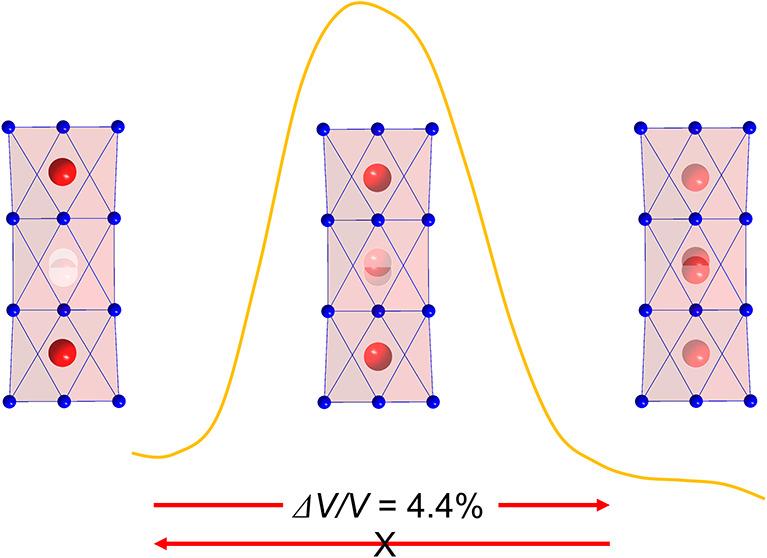

## 1 Introduction

Dense inorganic oxides are generally regarded as structurally rigid solids whose lattice responses are governed by anharmonic phonons and are therefore modest in magnitude.^1–3^ Large lattice changes typically arise only when symmetry or composition is altered, for example, through structural phase transitions, redox processes, or reconstructive transformations.^4–9^ Whether dense oxides can exhibit comparably large lattice responses through purely configurational relaxation, without symmetry breaking or compositional change, remains largely unexplored.

High-pressure synthesis provides a route to access nonequilibrium states in dense oxides that are stabilized under compression but retained upon recovery to ambient conditions.^10–14^ Beyond generating new crystal structures, this approach can imprint frustrated configurational landscapes composed of nearly degenerate atomic arrangements separated by substantial kinetic barriers. Such states may store latent configurational strain that can be released upon thermal activation, offering a fundamentally different pathway to structural response.

In the present work, we find that this framework provides a useful way to rationalize an unusual lattice response observed in a high-pressure phase. Specifically, a combination of competing local configurations, coupling between the cation distribution and lattice volume, and kinetic trapping of a high-density state appears to enable a large, symmetry-preserving lattice expansion through cation redistribution. We emphasize that this perspective is developed based on the experimental observations described below, rather than as a strictly predictive design strategy.

Here, we demonstrate this mechanism in the high-pressure-quenched dense oxide Ba_4_Ru_3_O_12_. The material exhibits a giant irreversible volumetric expansion of 4.4% between 450 and 650 K while retaining the crystallographic symmetry and oxygen stoichiometry. High-resolution synchrotron diffraction reveals cooperative redistribution of Ru within face-sharing RuO_6_ trimers, directly linking intratrimer cation configuration to lattice volume. Thermogravimetric, transport, and magnetic measurements exclude decomposition, redox processes, and electronic phase transitions, and first-principles calculations show that high-pressure synthesis stabilizes a frustrated configurational landscape that relaxes upon heating through intratrimer cation exchange. These results identify symmetry-preserving configurational cation redistribution as a mechanism for large lattice responses in dense oxides.

## 2 Experimental Section

Ba_4_Ru_3_O_12_ was synthesized in a multianvil press at 6 GPa and 1373 K for 30 min, followed by quenching to room temperature prior to decompression, according to established high-pressure synthesis procedures.^10,11^ All measurements were performed on the recovered material at ambient pressure. High-resolution synchrotron powder X-ray diffraction data were collected at BL02B2 at SPring-8. The sample was first cooled from room temperature to 100 K without collecting diffraction data, and diffraction patterns were subsequently collected upon heating from 100 to 800 K. Magnetization, electrical transport, thermogravimetric, and calorimetric measurements were carried out using standard techniques. Density functional theory calculations were performed using the projector augmented-wave method within the generalized gradient approximation of Perdew–Burke–Ernzerhof (PBE), as implemented in the Vienna ab initio Simulation Package (VASP).^15–18^ Minimum-energy paths for intratrimer Ru-site exchange were determined using the climbing-image nudged elastic band method.^19,20^ Further experimental and computational details are provided in the Supporting Information.

## 3 Results and Discussion

### 3.1 Structural Refinement and Temperature-Dependent Evolution

The crystal structure of Ba_4_Ru_3_O_12_ was investigated by using high-resolution synchrotron powder X-ray diffraction data collected upon heating from 100 to 800 K. Because this phase has not been previously reported, structure solution and model selection were carried out prior to final Rietveld refinement. The diffraction pattern was first indexed with rhombohedral lattice parameters, and candidate structural models were examined on the basis of systematic absences and related hexagonal perovskite-derived structures containing face-sharing octahedral trimers. During this process, a non-centrosymmetric *R*3̅*m* model and several centrosymmetric *R*3̅*m* models with similar lattice parameters were tested. A Ba_4_Re_2_CoO_12_-type model^21^ did not satisfactorily reproduce the observed intensities, whereas a BaIr_0.5_Co_0.5_O_3_-type model^22^ provided a more suitable starting framework. Further refinement of the atomic coordinates, Ru-site occupancies, and displacement parameters led to the final *R*3̅*m* model used in this work. Diffraction patterns at 450 K and above are well-described by this rhombohedral *R*3̅*m* model, consistent with a perovskite-derived framework comprising face-sharing RuO_6_ trimers. All reflections can be indexed without detectable impurity phases, and the refinements yield satisfactory agreement factors, supporting the proposed structural model (Table 1 and Figure 1).

**Table 1:** Refined Atomic Coordinates, Site Occupancies, and Isotropic Atomic Displacement Parameters for Ba_4_Ru_3_O_12_ at 450 and 650 K^a^

Atom	Wyckoff	*g*	*X*	*y*	*z*	*B* (Å^2^)
*T* = 450 K						
Ba1	6c	1	0	0	0.2096(1)	0.92(2)
Ba2	6c	1	0	0	0.3711(1)	1.27(3)
Ru1	6c	0.873(4)	0	0	0.0825(1)	0.62(4)
Ru2	6c	0.070(2)	0	0	0.0099(7)	0.24(30)
Ru3	3b	1	0	0	0.5	0.57(5)
O1	18 h	1	0.4971(6)	–*x*	0.2080(2)	1.21(11)
O2	18 h	1	0.5133(6)	–*x*	0.3803(2)	1.19(11)
*T* = 650 K						
Ba1	6c	1	0	0	0.2121(1)	2.26(3)
Ba2	6c	1	0	0	0.3737(1)	1.60(2)
Ru1	6c	0.509(3)	0	0	0.0890(1)	0.49(7)
Ru2	6c	0.432(2)	0	0	0.0047(3)	1.46(7)
Ru3	3b	1	0	0	0.5	1.00(4)
O1	18 h	1	0.5007(7)	–*x*	0.2056(3)	2.27(15)
O2	18 h	1	0.5131(6)	–*x*	0.3759(2)	1.81(14)

a Refinements were performed in the space group *R*3̅*m* (No. 166) with *Z* = 3 using synchrotron powder X-ray diffraction data collected at BL02B2, SPring-8 (λ = 0.6205853 Å). The refined occupancies correspond to the composition Ba_4_Ru_2.88_O_12_. The occupancies of the Ru1 and Ru2 sites were refined freely, without constraints, together with their isotropic displacement parameters. Structural and refinement parameters are as follows: 450 K, *a* = 5.73378(2) Å, *c* = 27.9222(2) Å, *V* = 794.993(7) Å^3^, *R*_wp_ = 10.62%, *R*_p_ = 7.45%, *R*_e_ = 4.40%, *R*_I_ = 3.94%, *R*_F_ = 2.66%; 650 K, *a* = 5.80731(2) Å, *c* = 28.4062(1) Å, *V* = 829.648(5) Å^3^, *R*_wp_ = 10.12%, *R*_p_ = 7.41%, *R*_e_ = 4.40%, *R*_I_ = 5.44%, *R*_F_ = 5.85%.

**Figure 1:**
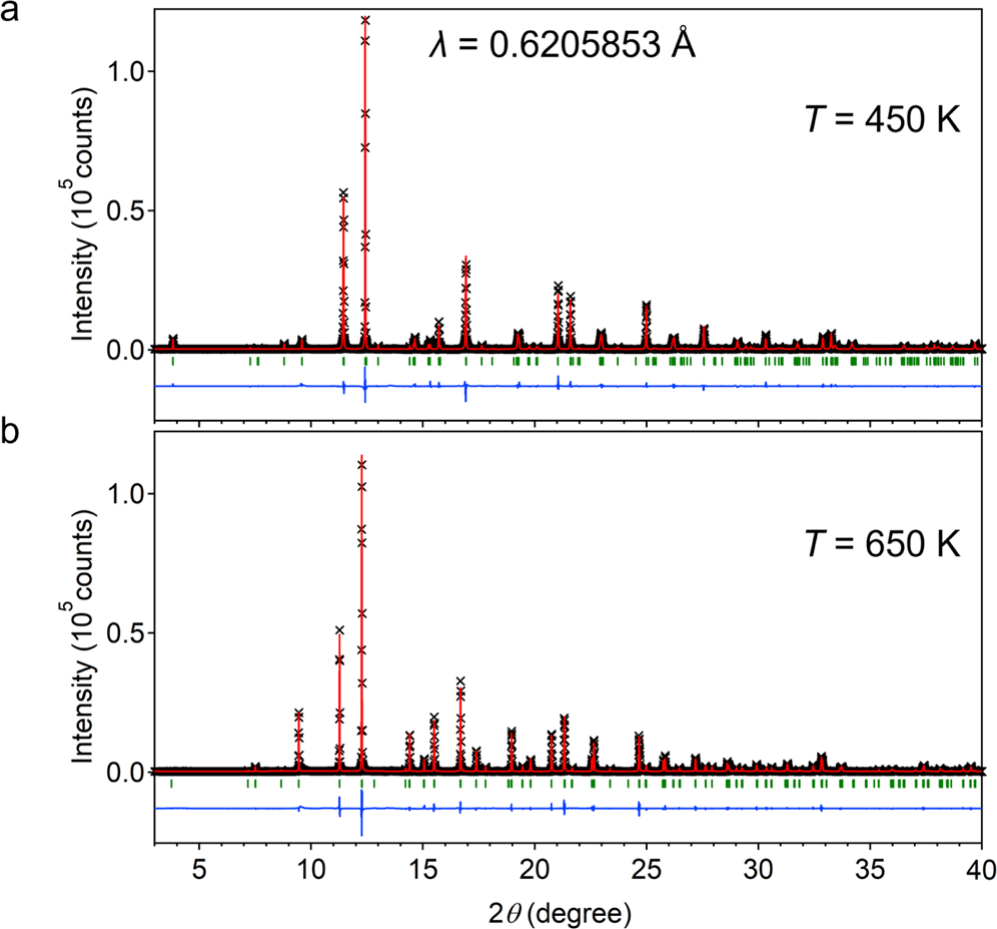
Rietveld refinements of synchrotron powder X-ray diffraction patterns of Ba_4_Ru_3_O_12_ collected at (a) 450 K and (b) 650 K. Black crosses represent the observed data, the red lines represent the calculated profiles, and the blue lines represent the difference curves. Green tick marks denote the allowed Bragg reflections of the *R*3̅*m* phase. The refined atomic parameters are summarized in Table 1. The incident X-ray wavelength was λ = 0.6205853 Å for both measurements.

The lattice parameters exhibit a pronounced, nonlinear temperature dependence (Figure 2a). In particular, both *a* and *c* increase significantly between 450 and 650 K, resulting in a substantial expansion of the unit-cell volume (Figure 2b). No additional reflections or peak splittings are observed in this temperature range (Figure S1), indicating that the expansion occurs without a crystallographic phase transition. The retention of *R*3̅*m* symmetry throughout this regime establishes that the lattice response is symmetry-preserving. The cooling data shown as open symbols in Figures 2a,b were obtained in a separate experiment conducted under identical temperature protocols and measurement conditions and are included for comparison.

**Figure 2:**
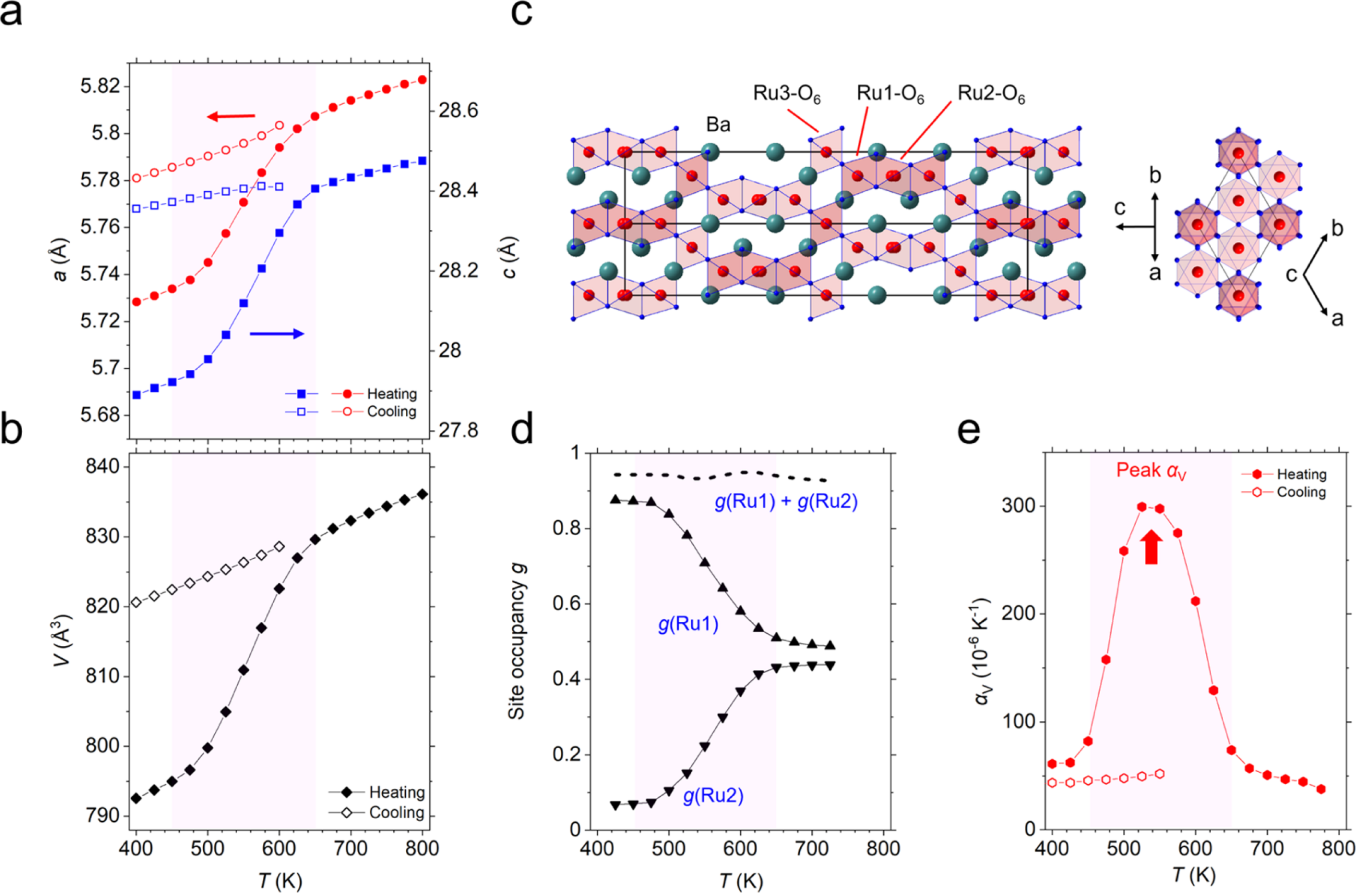
Symmetry-preserving intratrimer redistribution in Ba_4_Ru_3_O_12_ across the irreversible expansion. (a) Temperature dependence of lattice parameters a and c obtained from synchrotron powder X-ray diffraction during heating and cooling; the anomalous regime is highlighted. (b) Unit-cell volume *V* showing an irreversible increase over the same temperature range, with retention of the expanded state upon cooling. (c) Crystal structure of Ba_4_Ru_3_O_12_, highlighting the face-sharing RuO_6_ trimers (Ru_3_O_12_ units) and the Ru1 and Ru2 sites involved in intratrimer redistribution. (d) Refined site occupancies *g*(Ru1), *g*(Ru2), and their sum, demonstrating cooperative intratrimer site exchange at conserved total occupancy. (e) Volumetric thermal expansion coefficient (α_V_) derived from the refined lattice parameters; the maximum coincides with the occupancy redistribution regime, linking the macroscopic expansion to intratrimer rearrangement. Refinements were performed using Ba_4_Ru_3−δ_O_12_ with δ ≈ 0.12; SEM–EDX and TGA measurements are consistent with the refined composition and constant oxygen content (Supporting Information). Error bars are comparable to or smaller than the symbol size.

To probe the microscopic origin of this structural evolution, the occupancies of the Ru1 and Ru2 sites within the Ru_3_O_12_ trimers (Figure 2c), together with their isotropic displacement parameters, were refined without constraints. The results reveal a systematic temperature-dependent redistribution of Ru between the two crystallographic sites. Importantly, the total occupancy, *g*(Ru1) + *g*(Ru2), remains conserved within experimental uncertainty across the entire temperature range, consistent with intratrimer site exchange at a fixed stoichiometry rather than compositional variation.

Although site occupancies and atomic displacement parameters are commonly correlated in Rietveld analysis, several independent observations support the reliability of the refined occupancies. The total Ru occupancy remains conserved within experimental uncertainty (Figure 2d), the occupancies evolve smoothly and monotonically with temperature (Figure 6c), and the refined compositions agree with independent chemical analyses and thermogravimetric measurements (Figures S3 and S4). No clear evidence of decomposition or oxygen loss was observed within the investigated temperature range, as confirmed by thermogravimetric analysis and the absence of impurity reflections in the diffraction data. Together, these results indicate that the observed redistribution reflects an intrinsic structural evolution rather than refinement artifacts.

At temperatures of 425 K and below, the diffraction patterns deviate markedly from those observed at higher temperatures. Clear peak splitting is observed at 100 K, indicating symmetry lowering relative to that of the *R*3̅*m* structure. The splitting is consistent with a lower-symmetry metric, tentatively triclinic, although a definitive structure determination could not be obtained from powder diffraction data alone. Several reflections also exhibit anisotropic broadening and shoulder-like features, suggesting local disorder and/or static displacements of Ru ions within the face-sharing trimers.

Taken together, these results indicate that the as-recovered material adopts a locally disordered state at low temperature. Upon heating to 450–650 K, this state evolves into a well-defined average structure with preserved symmetry and redistributed cation occupancies, which underpins the anomalous lattice expansion described below. Under ambient-pressure synthesis conditions, the related lower-oxygen compound Ba_4_Ru_3_O_10_ is known to form,^23,24^ whereas Ba_4_Ru_3_O_12_ with the high formal Ru valence is obtained only under high-pressure conditions in the present study. To our knowledge, Ba_4_Ru_3_O_12_ has not been reported from ambient-pressure synthesis.

### 3.2 Giant Irreversible Lattice Expansion in a Dense Oxide

Ba_4_Ru_3_O_12_ exhibits a pronounced and irreversible volumetric expansion of 4.4% between 450 and 650 K (Figure 2). The volumetric thermal expansion coefficient α_V_ displays a sharp maximum near 550 K, coincident with the most rapid increase in unit-cell volume. The expanded state is retained upon cooling, and subsequent thermal cycles reproduce the enlarged volume, confirming the irreversibility of the transformation.

No additional symmetry-breaking reflections emerge during heating (Figure S1), indicating that the expansion proceeds without a symmetry-lowering phase transition but rather through a largely continuous, isosymmetric transformation within the experimental resolution, involving changes in site occupancies. Such behavior is highly unusual for dense crystalline solids, in which large volume changes are typically associated with structural transformations. Although isosymmetric transitions with large volume changes have been reported in systems such as CaFe_2_As_2_,^25^ these are typically first-order transitions involving discontinuous changes and phase coexistence. By contrast, the present system exhibits a largely continuous, symmetry-preserving evolution without a detectable discontinuity or phase coexistence.

To place this response in context, the thermal expansion of Ba_4_Ru_3_O_12_ was compared with representative crystalline materials that remain chemically and structurally stable above 400 K (Figures 3 and S7). Whereas dense oxides generally exhibit modest thermal expansion governed by lattice anharmonicity, Ba_4_Ru_3_O_12_ exceeds representative oxide, alloy, and salt benchmarks within this temperature range. In most crystalline solids, comparably large expansion anomalies accompany reconstructive or symmetry-breaking transformations (e.g., Ca-substituted PbCrO_3_,^26^ NaSbF_6_,^27^ RbNbO_3_,^28,29^ and CsPbI_3_ ^30^) or coincide with electronic or magnetic phase transitions.^5,6,8,9^ By contrast, Ba_4_Ru_3_O_12_ shows a large irreversible expansion without detectable changes in crystallographic symmetry or overall composition.

**Figure 3:**
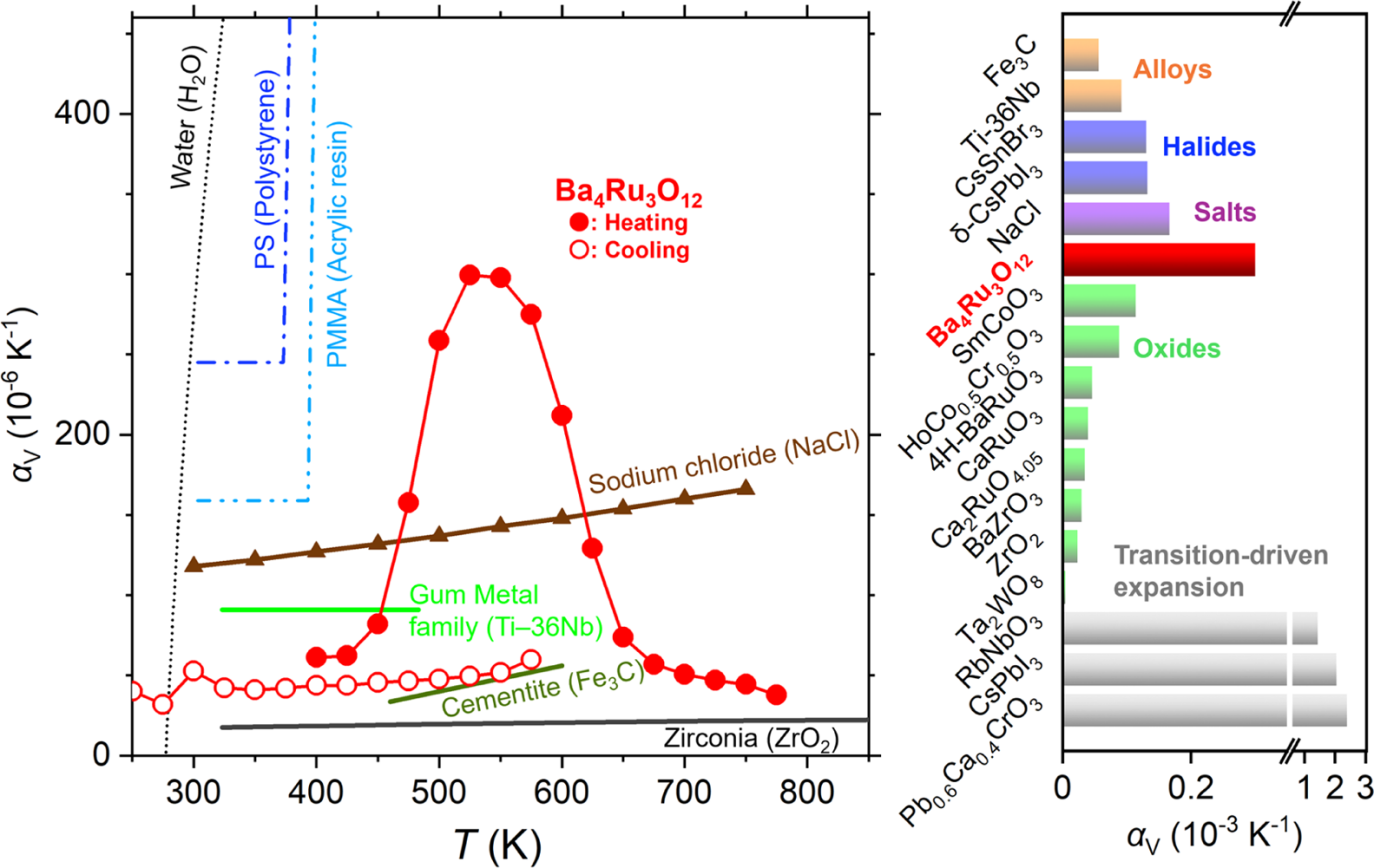
Benchmarking the thermal expansion of Ba_4_Ru_3_O_12_ above 400 K. Temperature-dependent volumetric thermal expansion coefficient (α_V_) of Ba_4_Ru_3_O_12_ compared with representative crystalline materials, including oxides, salts, and alloys; soft materials are shown for context. For Ba_4_Ru_3_O_12_, both the initial-heating and subsequent-cooling thermal expansion coefficients are shown to distinguish the irreversible expansion from the reversible response of the expanded state. The bar chart summarizes the maximum α_V_ values reported above 400 K for materials that remain chemically and structurally stable within the corresponding measurement ranges. In comparison with these benchmark materials, Ba_4_Ru_3_O_12_ shows an unusually large α_V_ among dense oxides and an irreversible volume increase without detectable changes in overall composition or crystallographic symmetry. The underlying data set and references are provided in Figure S7, and the inclusion and exclusion criteria are described in Section S7.

This combination of large magnitude, irreversibility, and structural integrity points to a mechanism distinct from conventional transition-driven lattice anomalies as explored below. The thermal expansion coefficient obtained upon cooling from the expanded high-temperature state is much smaller than that observed during the initial heating process (Figure 3), confirming that the giant expansion is associated with the irreversible transformation of the metastable state rather than with ordinary reversible thermal expansion.

### 3.3 Microscopic Origin: Intratrimer Redistribution and Frustration Release

To elucidate the mechanism underlying the anomalous lattice expansion, we examined the temperature-dependent redistribution of Ru within the face-sharing Ru_3_O_12_ trimers (Figure 2c). Upon heating, the refined occupancies *g*(Ru1) and *g*(Ru2) evolved cooperatively (Figure 2d): at 450 K, *g*(Ru1) = 0.873(4) and *g*(Ru2) = 0.070(2), whereas at 650 K, *g*(Ru1) = 0.509(3) and *g*(Ru2) = 0.432(2) (Table 1). The total occupancy remains conserved within uncertainty, indicating intratrimer site exchange at a fixed stoichiometry rather than Ru loss, phase separation, or defect formation.

The maximum in α_V_ coincides with this redistribution regime (Figure 2e), directly linking the macroscopic volume expansion to intratrimer rearrangement. Temperature-dependent refinements of structural parameters show continuous evolution across this temperature range (Figure S2), with no evidence of a reconstructive transformation.

These observations support a mechanism in which the low-temperature state stores structural frustration within the face-sharing trimers. Upon heating, this frustration is gradually released through the symmetry-preserving redistribution of Ru, enabling cooperative lattice expansion through a largely continuous, symmetry-preserving (isosymmetric) transformation within the experimental resolution. This mechanism provides a unified explanation for the large, irreversible, and structurally intact volume increase observed in Ba_4_Ru_3_O_12_.

This behavior differs from conventional expansion phenomena driven by structural phase transitions, redox processes, or defect formation, highlighting structural frustration as an internal degree of freedom governing the lattice response in dense oxides.

### 3.4 Exclusion of Redox, Decomposition, and Electronic Transitions

Thermogravimetric analysis under flowing N_2_ shows no detectable mass change up to 700 K (Figure S4b), indicating the absence of decomposition or oxygen loss under inert conditions. Under a reducing atmosphere, a controlled mass loss consistent with oxygen removal is observed (Figure S4c), confirming the initial oxygen stoichiometry. SEM–EDX measurements before and after thermal cycling reveal unchanged Ba/Ru ratios (Figure S3), consistent with the refined composition Ba_4_Ru_3−δ_O_12_ (Table 1). These results indicate that the observed expansion occurs without measurable compositional modification.

Electrical resistivity and magnetic susceptibility evolve smoothly across 450–650 K (Figure 4a–d), without anomalies indicative of electronic or magnetic phase transitions. Although the low-temperature state evolves upon repeated cycling (Figures 4c and S5), no discontinuity coincides with the expansion regime. This behavior contrasts with systems such as Ba_4_Ru_3_O_10_,^7^ the Verwey transition in Fe_3_O_4_,^8^ and charge ordering in CaFeO_3_,^9^ where structural anomalies are coupled to charge or magnetic ordering transitions.

**Figure 4:**
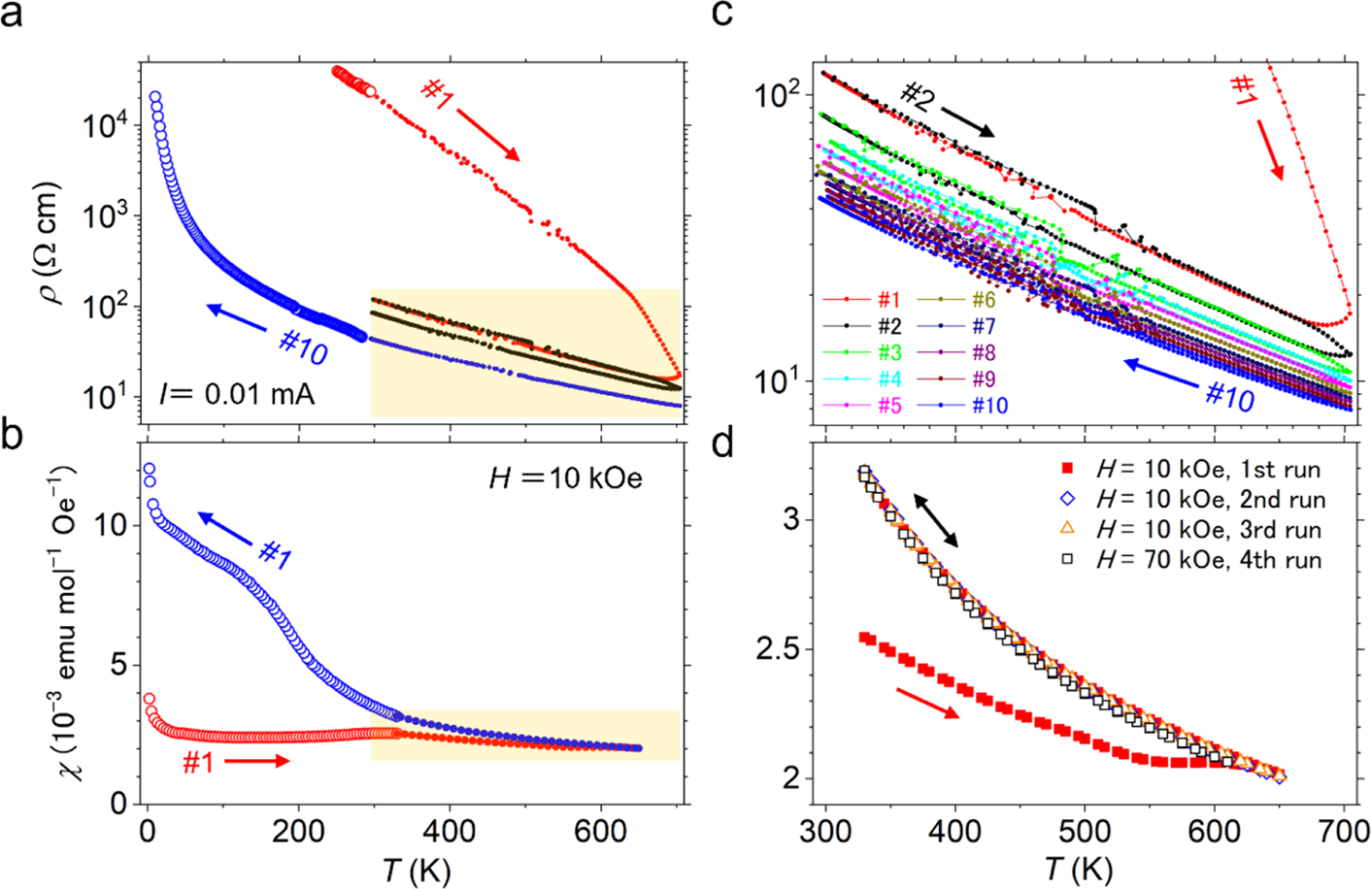
Transport and magnetic properties of Ba_4_Ru_3_O_12_ during thermal cycling. (a) Electrical resistivity, ρ­(*T*), measured during the first heating run (run 1) and after repeated cycling (run 10) under a dc current of *I* = 0.01 mA. (b) dc magnetic susceptibility, χ­(*T*), measured at *H* = 10 kOe during the first run. The shaded regions in (a,b) indicate the temperature ranges enlarged in panels (c,d), respectively. (c) Expanded view of ρ­(*T*) over consecutive heating and cooling cycles (runs 1–10), showing evolution of the low-temperature state while remaining continuous through the 450–650 K expansion regime. (d) χ­(*T*) between 330 and 650 K measured at *H* = 10 and 70 kOe over successive runs, showing only modest thermal hysteresis and no abrupt anomaly within the expansion regime. The smooth evolution of ρ­(*T*) and χ­(*T*) across the expansion window supports the absence of a discrete electronic or magnetic phase transition and is consistent with a structurally driven relaxation process.

Consistent with these observations, differential scanning calorimetry reveals a thermal event during the first heating cycle that is largely suppressed upon reheating (Figure S4a), in line with the irreversible nature of the expansion. Together, these results exclude decomposition, redox processes, and electronic or magnetic phase transitions as the primary drivers of lattice expansion, supporting a chemically intact structural relaxation mechanism.

### 3.5 Frustrated Configurational Landscape at Ambient Pressure

To elucidate the origin of the irreversible relaxation, we examined the configurational energetics using density functional theory.^15–18^ At zero pressure, distinct Ru-site arrangements form a dense manifold of competing states: several configurations lie within approximately 50 meV per formula unit of the lowest-energy structure, spanning an overall energy range of about 0.4 eV per formula unit (Figure 5a–c). Notably, multiple inequivalent configurations exhibit similar *g*(Ru1) values yet differ in total energy (Figure 5c), indicating that site occupancy alone does not uniquely determine stability. This near-degeneracy points to a frustrated configurational landscape within the face-sharing Ru_3_O_12_ trimer sublattice.

**Figure 5:**
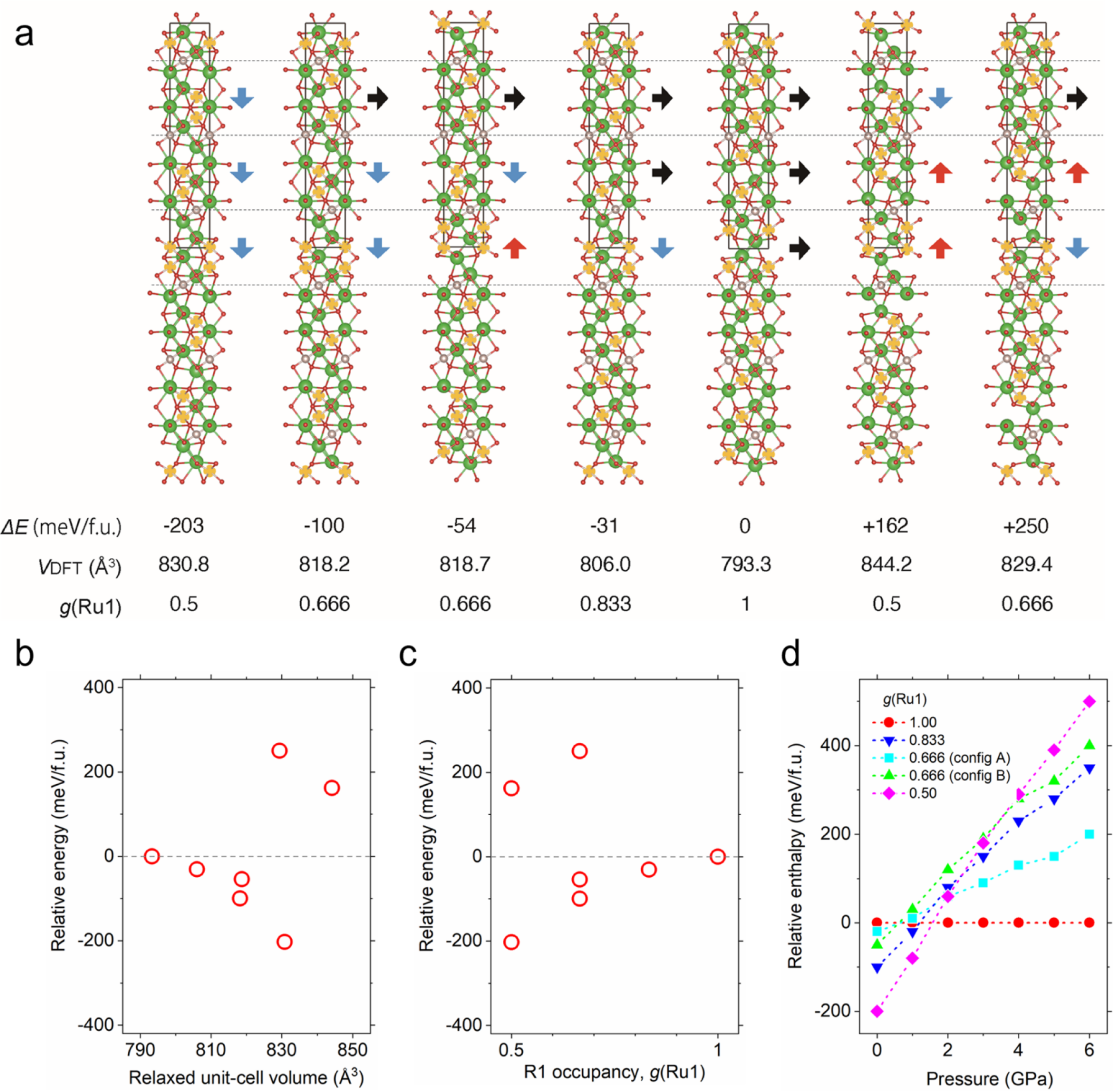
Theoretical support for pressure-stabilized metastability and intratrimer Ru-site rearrangement in Ba_4_Ru_3_O_12_. (a) DFT-relaxed structures for representative Ru-site configurations at zero pressure, illustrating distinct Ru distributions within the face-sharing Ru_3_O_12_ trimers. Values below each structure indicate the relative energy, Δ*E* (meV per formula unit, referenced to the lowest-energy configuration), the relaxed unit-cell volume, *V* (Å^3^), and the Ru1 site occupancy, *g*(Ru1). Arrows denote representative directions of intratrimer Ru-site redistribution. (b) Relative energy as a function of relaxed unit-cell volume for the same configurations, revealing a range of equilibrium volumes within the low-energy manifold at zero pressure. (c) Relative energy as a function of *g*(Ru1), showing that inequivalent configurations may have similar occupancies but different energies. (d) Pressure-dependent relative enthalpy per formula unit for representative configurations indexed by *g*(Ru1), demonstrating that compression lifts the quasi-degeneracy of the low-pressure manifold and stabilizes the fully ordered state with *g*(Ru1) = 1.00. These results support a picture in which high-pressure synthesis traps a configuration manifold that can relax through intratrimer Ru-site rearrangement after recovery to ambient pressure.

These configurations also show substantial variation in relaxed unit-cell volume (Figure 5a,b), revealing strong coupling between Ru-site distribution and lattice dimensions. Under applied pressure, the enthalpy hierarchy is markedly reorganized (Figure 5d): the quasi-degenerate manifold collapses, and the fully ordered configuration with *g*(Ru1) = 1.00 becomes energetically favored. This pressure-induced stabilization provides a thermodynamic basis for the high-pressure synthesis,^11–14^ and suggests that the sample recovered at ambient pressure retains a configurational state inherited from compression that relaxes upon heating through intratrimer Ru-site rearrangement.

### 3.6 Kinetic Gating and Direct Volume–Occupancy Coupling

The persistence of the as-recovered configuration at room temperature implies a substantial kinetic barrier to intratrimer redistribution. Climbing-image nudged elastic band calculations^19,20^ yield an activation barrier *E*_a_ of approximately 2.0 eV for representative Ru-site exchange pathways (Figure 6a). Arrhenius analysis based on these barriers (Figure 6b) indicates that redistribution becomes accessible on laboratory time scales within 450–650 K while remaining effectively frozen at ambient temperature. The experimentally observed temperature window of irreversible expansion is therefore consistent with the calculated activation scale.

**Figure 6:**
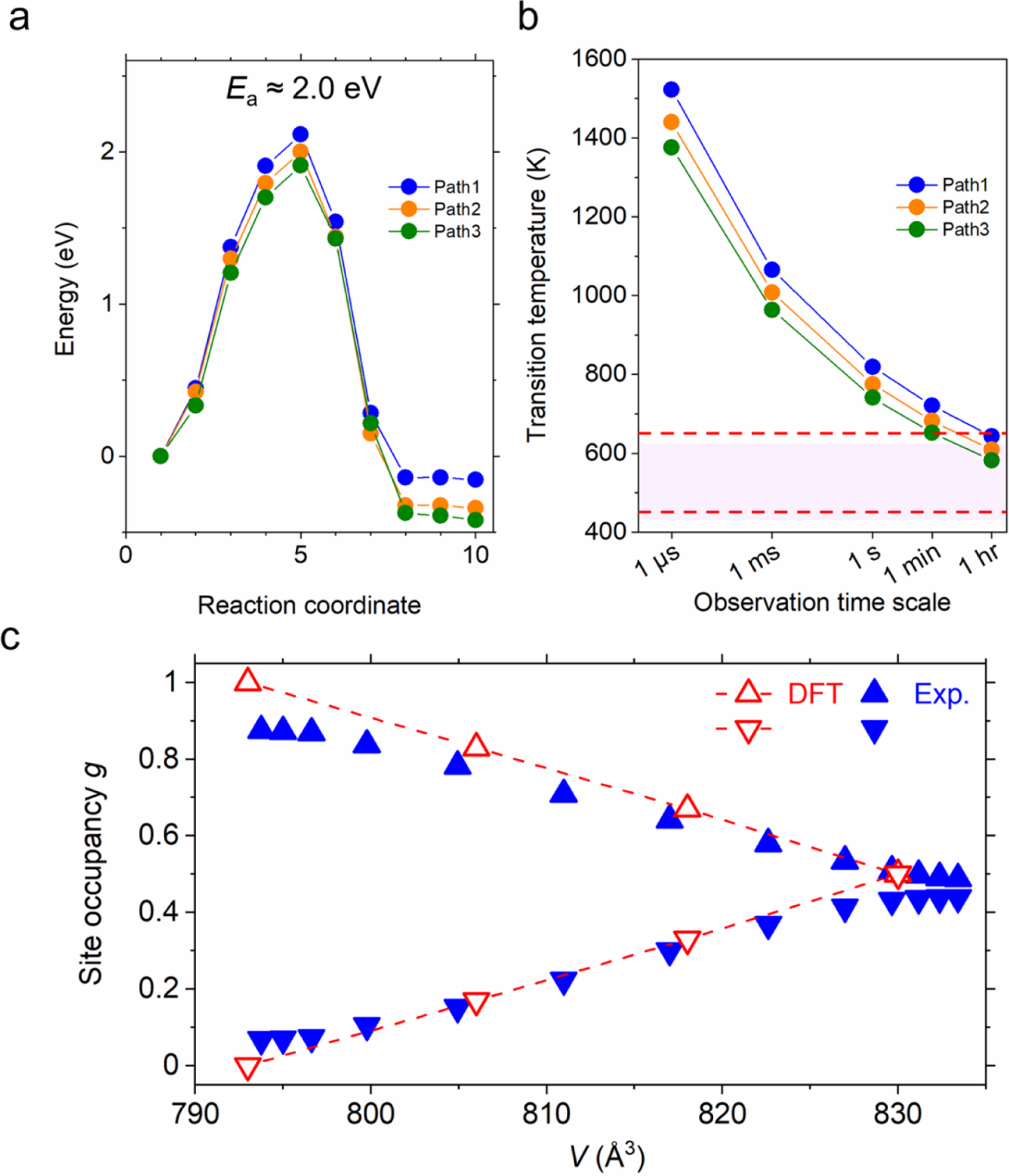
Kinetic accessibility and direct volume-occupancy coupling of intratrimer Ru-site redistribution in Ba_4_Ru_3_O_12_. (a) Climbing-image nudged elastic band (CI-NEB) minimum–energy profiles for three representative Ru-site exchange pathways. Path 1, Path 2, and Path 3 correspond to stepwise reductions in Ru1-site occupancy from 1.00 to 0.833, from 0.833 to 0.666, and from 0.666 to 0.50, respectively, accompanied by corresponding changes in the local Ru arrangements within the Ru_3_O_12_ trimers. Energies are referenced to the initial configuration of each path. The corresponding activation barriers are all approximately 2.0 eV. (b) Characteristic transition temperature estimated from Arrhenius analysis as a function of observation time scale for the three exchange pathways. The shaded region indicates the experimental temperature range (450–650 K) of the irreversible volume expansion. (c) Ru-site occupancy, *g*, plotted against unit-cell volume, *V*. Experimental data (filled symbols) and DFT results (open symbols) follow the same near-linear relation, establishing a direct link between configurational relaxation and macroscopic lattice expansion.

Importantly, both experiment and density functional theory reveal a linear correlation between Ru site occupancy and unit cell volume (Figure 6c). The calculated structures follow the same trend as the experimental data, demonstrating that the macroscopic lattice expansion is directly coupled to the intratrimer redistribution rather than arising from an indirect electronic effect.

### 3.7 Mechanistic Picture

Taken together, these results support a frustration-mediated relaxation mechanism established by the high-pressure synthesis. Under compression, an ordered configuration is thermodynamically stabilized (Figure 5d). Upon decompression to ambient pressure, a quasi-degenerate frustrated manifold emerges (Figures 5a–c), yet redistribution among these configurations remains kinetically inhibited at low temperature by a substantial activation barrier (Figures 6a and S6). Heating into the 450–650 K range overcomes this barrier, enabling cooperative intratrimer Ru-site exchange (Figure 2d) and releasing stored configurational strain. The equilibrium volume correspondingly increases (Figures 2b and 6c), and the expanded configuration is retained upon cooling (Figure 7), resulting in irreversible structural relaxation without redox processes, mass loss, or symmetry breaking.

**Figure 7:**
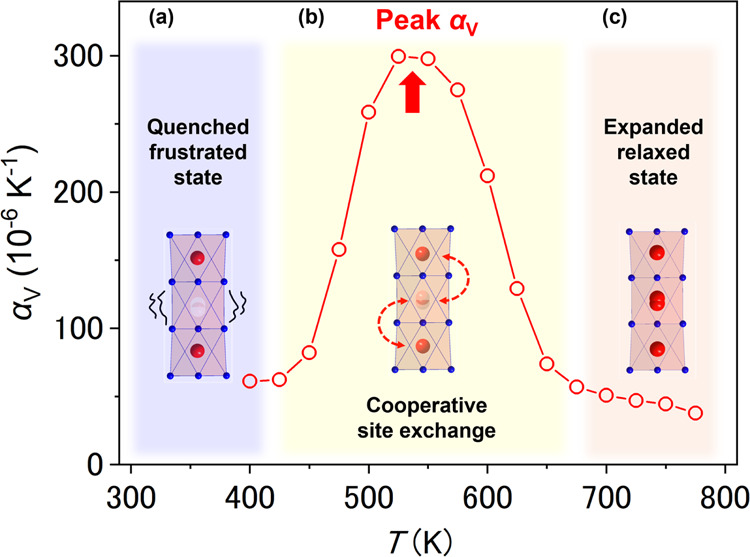
Irreversible thermal expansion in Ba_4_Ru_3_O_12_ driven by frustration release. The volumetric thermal expansion coefficient, α_V_, exhibits a pronounced maximum between 450 and 650 K, corresponding to a net volumetric expansion of 4.4% over the same temperature range. The unit-cell volume increases monotonically without an accompanying change in crystallographic symmetry. Schematics illustrate (a) a quenched frustrated room-temperature state with multiple nearly degenerate cation configurations in the Ru_3_O_12_ trimers, (b) cooperative intratrimer Ru-site exchange upon heating, and (c) the expanded relaxed state stabilized after frustration release.

Although the detailed atomic structure of the as-recovered low-temperature state could not be uniquely resolved from powder diffraction data alone, the combined diffraction, refinement, thermal, and theoretical results support a locally disordered precursor that relaxes upon heating. This mechanism differs fundamentally from redox-driven lattice anomalies,^7–9^ martensitic or precipitation-mediated expansion in alloys,^31,32^ and classical anharmonic thermal expansion in dense oxides.^1–3^

## 4 Conclusion

We demonstrate that a dense oxide synthesized under high pressure can retain a metastable, low-volume cation configuration that relaxes upon heating through intratrimer cation redistribution, producing a large and irreversible lattice expansion while preserving both the crystallographic symmetry and chemical composition. In Ba_4_Ru_3_O_12_, this transformation reflects the release of a kinetically trapped cation configuration formed under compression, providing a simple physical picture of the observed behavior.

More broadly, our results suggest that similar lattice responses may be realized in systems that combine (i) multiple nearly degenerate cation configurations, (ii) strong coupling between cation arrangement and lattice volume, and (iii) kinetic barriers that enable metastable states to be retained after high-pressure synthesis. These features—supported by first-principles calculations in the present system—identify a general route for achieving large structural responses in dense solids without compositional change or symmetry breaking. Dense oxides prepared far from equilibrium thus emerge as a promising platform for harnessing configurational degrees of freedom as a functional handle for controlling lattice properties.

## Supplementary Material



## Data Availability

Source data are provided with this paper. All data supporting the findings of this study are available within the article and its Supporting Information. Additional data, including refined structural models and raw diffraction files, are available from the corresponding author upon reasonable request.

## Notes

Experimental methods; structural refinement and analysis; SEM and EDX composition analysis; thermal and chemical stability data; magnetic and transport measurements; NEB kinetic analysis; and benchmark compilation methodology (PDF)
