# Supplementary material for: Giant Lattice Expansion through Structural Frustration Release in a Dense Oxide
Source: J Am Chem Soc. 2026 May 20;148(21):22380–8. doi: 10.1021/jacs.6c07579 (PMC13244468; doi:10.1021/jacs.6c07579)
Supplement: Supplementary file 1 [file ja6c07579_si_001.pdf]

Supporting Information

Giant Lattice Expansion through Structural Frustration Release in a Dense Oxide

Zhijun Li,[1,2] Hongbo Yuan,[1,2] Alexei A. Belik,[1] Terumasa Tadano,[3] Yoshihiro Tsujimoto,[1,2] Kazunari Yamaura,[1,2,*]

[1] Research Center for Materials Nanoarchitectonics (MANA), National Institute for Materials Science, 1-1 Namiki, Tsukuba, Ibaraki 305-0044, Japan

[2] Graduate School of Chemical Sciences and Engineering, Hokkaido University, North 10 West 8, Kita-ku, Sapporo, Hokkaido 060-0810, Japan

[3] Research Center for Magnetic and Spintronic Materials (CMSM), National Institute for Materials Science, 1-2-1 Sengen, Tsukuba, Ibaraki 305-0047, Japan

[*]Correspondence and requests for materials should be addressed to K.Y. (email: YAMAURA.Kazunari@nims.go.jp).

Table of Contents

S1. Experimental Methods
S2. Structural Refinement and Analysis
S3. Composition and Valence Analysis
S4. Thermal and Chemical Stability
S5. Magnetic and Transport Behavior
S6. Kinetic Analysis from NEB
S7. Benchmark Compilation Methodology
Supplementary References

The Supporting Information provides detailed experimental procedures, structural refinements, and additional analyses supporting the results presented in the main text.

S1. Experimental Methods

Materials Synthesis: $Ba_4Ru_3O_{12}$ was synthesized under high-pressure, high-temperature conditions. BaO_2 (99%, Japan Pure Chemical Co., Ltd.), RuO_2 (99.9%, Rare Metallic Co., Ltd.), and Ru (99.9%, Strem Chemicals Inc.) were weighed, mixed in an Ar-filled glovebox, and sealed in Pt capsules. The reaction was carried out at 6 GPa and 1373 K for 30 min using a multi-anvil press (CTF-MA1500P, C&T Factory, Tokyo, Japan). Samples were quenched to room temperature prior to decompression over approximately 120 min, and the recovered products were lightly ground for characterization. All measurements in this study were performed on the material recovered at ambient pressure.

Several nominal Ba/Ru ratios (1.25–2.00) were examined; a starting ratio of Ba/Ru = 1.5 yielded the lowest impurity level according to laboratory powder X-ray diffraction (Rigaku MiniFlex, Cu K_α radiation). The product composition determined by energy-dispersive X-ray spectroscopy (EDX) and synchrotron Rietveld refinement corresponds to Ba/Ru ≈ 1.39 (see below). The refined composition is $Ba_4Ru_{3-\delta}O_{12}$ with $\delta \approx 0.12$; for simplicity, this material is referred to as $Ba_4Ru_3O_{12}$ throughout the manuscript. The discrepancy between the nominal and refined Ba/Ru ratios remains unresolved but may arise from minor Ba-containing amorphous phases (e.g., BaO) formed during synthesis or amorphized upon recovery to ambient conditions. The nominal oxygen content was further adjusted by varying the Ru/RuO_2 starting ratio (up to ~3 mol% nominal excess oxygen), but no detectable changes in the diffraction patterns were observed.

Synchrotron X-ray Diffraction: High-resolution synchrotron powder X-ray diffraction was performed at beamline BL02B2 (SPring-8) using a Debye–Scherrer camera (goniometer radius 286.48 mm).[1] Data were collected between 100 and 800 K on heating using a nitrogen gas-blowing system for temperature control. Cooling data were obtained in a separate experiment in which the sample was heated to a maximum temperature of 675 K prior to cooling, as partial decomposition was observed above ~750 K. Diffraction patterns were recorded at selected temperatures in 25 K steps, with each measurement completed within approximately 3 min per temperature point (including acquisition). The X-ray wavelength (λ = 0.6205853 Å) was calibrated using a CeO_2 standard. Finely ground powders were loaded into rotating Lindemann glass capillaries (0.2 mm inner diameter). Rietveld refinements were carried out using RIETAN-VENUS.[2–4]

Magnetic and Electrical Transport Measurements: Magnetization was measured using a Quantum Design MPMS3 SQUID magnetometer (Quantum Design Inc., USA) in dc magnetic fields up to 70

kOe and at temperatures up to 400 K. Temperature-dependent magnetization was recorded under zero-field-cooled (ZFC) and field-cooled (FC) protocols, and isothermal *M–H* loops were collected at selected temperatures. Measurements above 400 K were performed using the MPMS3 oven option. Pellets were mounted on the oven holder using Zircar cement and covered with Cu foil (0.003 mm thick) to improve thermal homogeneity. Measurements above 400 K were carried out under high vacuum, thereby excluding processes such as sample oxidation.

Electrical resistivity was measured using a standard four-probe method. Measurements up to 300 K were performed in a Quantum Design PPMS system using sintered pellets, with electrical contacts made using Ag epoxy. The epoxy was cured under vacuum at temperatures up to 420 K, below the structural transition range. High-temperature resistivity measurements (>300 K) were carried out using a laboratory-built setup equipped with a tabletop vacuum furnace (MILA-5000, ADVANCE RIKO Inc., Japan) with an applied current of 0.01 mA.

Scanning Electron Microscopy and Energy-Dispersive X-ray Spectroscopy: Scanning electron microscopy (SEM) was performed using a TM3000 microscope (Hitachi High-Technologies Corp., Japan). Elemental compositions were determined by energy-dispersive X-ray spectroscopy (EDX; SwiftED3000, Oxford Instruments, UK) operated at an accelerating voltage of 15 kV.

Two pellet specimens from the same batch were examined. One specimen was annealed in air at 700 K for 12 h and furnace-cooled, while the other was measured in the as-synthesized state. Prior to measurement, both specimens were polished using 0.3 μm alumina lapping film.

Thermal Analysis: Thermogravimetric analysis (TGA) was performed using a Rigaku TG-DTA8122 instrument under ≈9% H_2 in Ar (heated to 1073 K at 10 K min^{-1} with isothermal holds of up to 6 h) and under flowing N_2 (heated to 700 K at 5 K min^{-1}). Differential scanning calorimetry (DSC) was carried out using a DSC-60 Plus (Shimadzu, Japan) between 160 and 700 K at heating rates of 10, 20, 50, and 80 K min^{-1}. For each heating rate, two consecutive heating cycles were recorded, with controlled cooling to 160 K between the first and second cycles. A fresh sample from the same batch was used for each DSC measurement.

Computational Methods: Density functional theory (DFT) calculations were performed using the Vienna Ab initio Simulation Package (VASP).[5,6] The projector augmented-wave (PAW) method was employed,[7] using the recommended PAW potentials supplied with VASP. Exchange–correlation effects were treated within the generalized gradient approximation (GGA) using the Perdew–Burke–Ernzerhof (PBE) functional.[8]

A plane-wave energy cutoff of 550 eV was used. Brillouin-zone integrations were performed using an 8 × 8 × 1 Monkhorst–Pack k-point mesh.[9] Structural optimizations were carried out until the total-energy change between two consecutive ionic steps was smaller than 10^{-3} eV. Spin–orbit

coupling was not included. Minimum-energy pathways for Ru-site exchange were determined using the climbing-image nudged elastic band (CI-NEB) method.[10,11] Energies along the reaction coordinate were referenced to the initial state.

S2. Structural Refinement and Analysis

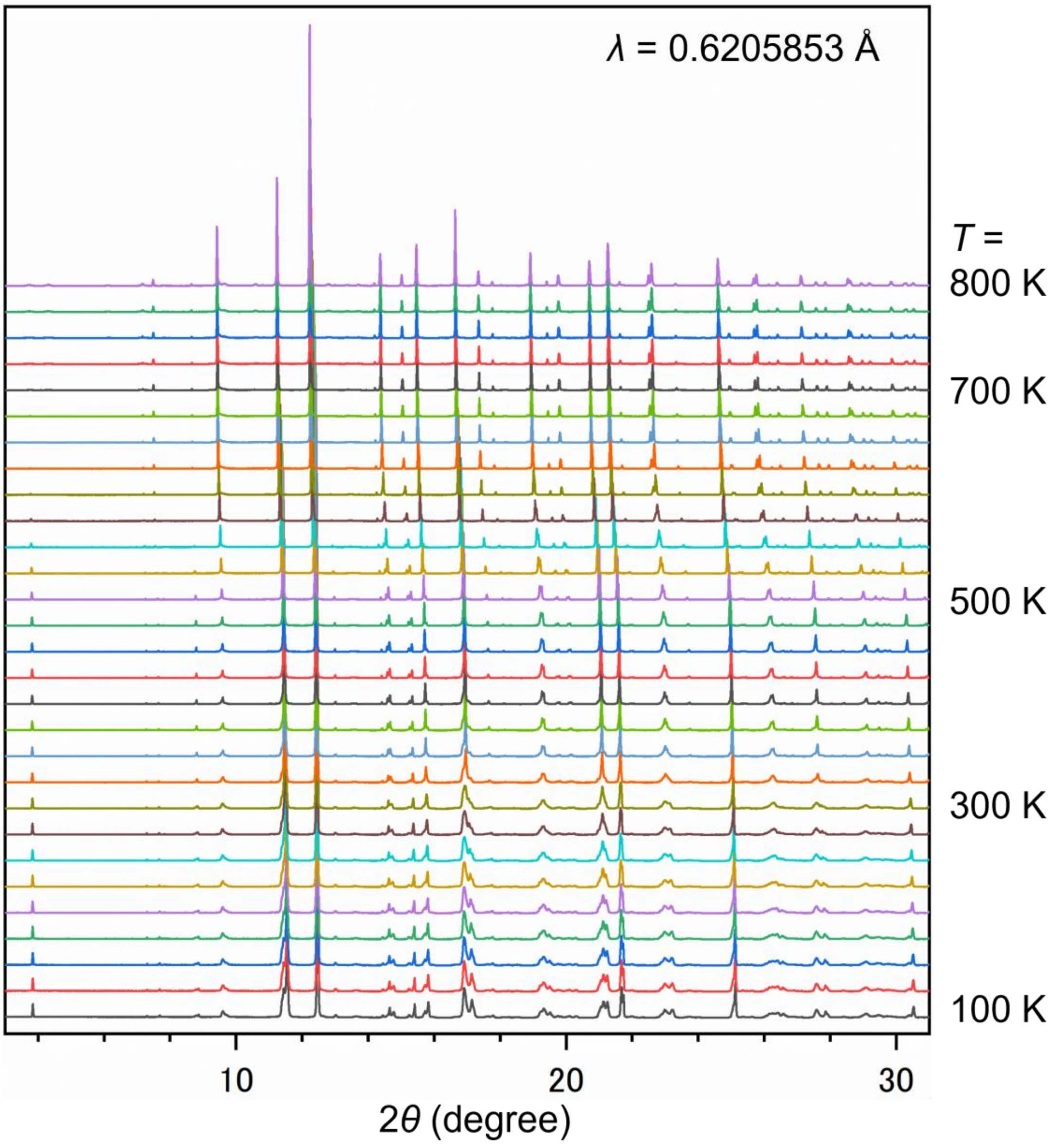

Fig. S1: Temperature-dependent synchrotron diffraction of $Ba_4Ru_3O_{12}$.

Stacked high-resolution synchrotron powder X-ray diffraction patterns (λ = 0.6205853 Å) collected during heating from 100 to 800 K in 25 K steps. Selected temperatures are indicated on the right. Progressive peak shifts and intensity changes reflect the structural evolution across the anomalous

450–650 K regime associated with the irreversible expansion. Above ~750 K, indications of partial decomposition appear. For example, at 800 K weak broadened reflections attributable to $BaCO_3$ and unidentified impurity phases are observed, together with broadening of some reflections of the main phase.

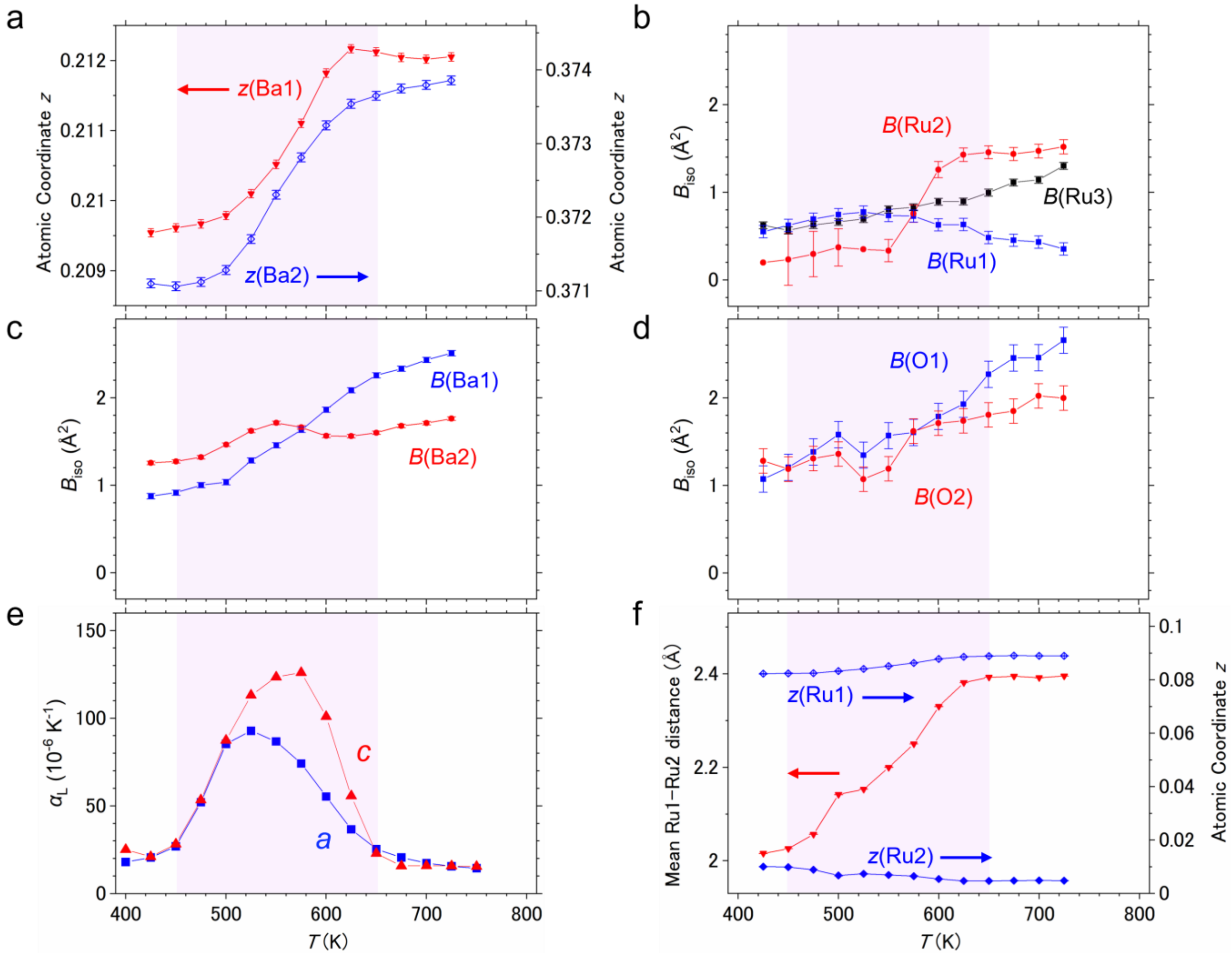

Fig. S2: Temperature-dependent refined structural parameters of $Ba_4Ru_3O_{12}$.

Structural parameters obtained from Rietveld refinement of synchrotron powder X-ray diffraction data as a function of temperature. (a) Fractional coordinates *z*(Ba1) and *z*(Ba2). (b) Isotropic displacement parameters *B*(Ru1), *B*(Ru2), and *B*(Ru3). Some points for *B*(Ru2) were fixed during refinement and are therefore plotted without error bars. (c) Isotropic displacement parameters *B*(Ba1) and *B*(Ba2). (d) Isotropic displacement parameters *B*(O1) and *B*(O2). (e) Linear thermal expansion coefficients α_L along the *a* and *c* axes derived from the lattice parameters. (f) Mean Ru1–Ru2 distance together with fractional coordinates *z*(Ru1) and *z*(Ru2). The shaded region (450–650 K) marks the anomalous structural regime associated with the irreversible expansion. Where not visible, error bars are comparable to or smaller than the marker size.

S3. Composition and Valence Analysis

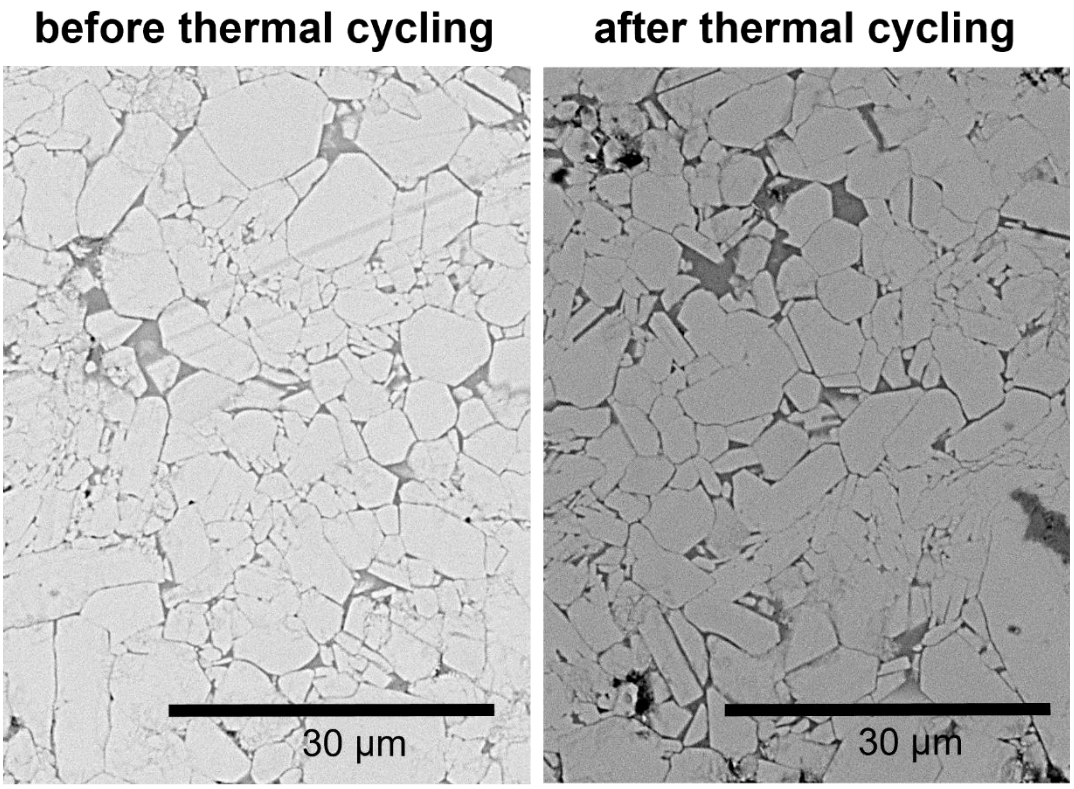

Fig. S3: SEM and EDX analysis of $Ba_4Ru_3O_{12}$ before and after thermal cycling.
Backscattered-electron SEM images of a sintered pellet before (left) and after (right) thermal cycling between 330 and 650 K (scale bars: 30 µm). No significant change in grain morphology is observed. EDX gives Ba/Ru = 1.36(2) before cycling and 1.35(5) after cycling, consistent with the refined composition $Ba_4Ru_{2.88}O_{12}$ (Ba/Ru = 1.39) and indicating no measurable compositional modification upon cycling.

Composition analysis: Rietveld refinements of the BL02B2 synchrotron data yield $Ba_4Ru_{3-\delta}O_{12}$ with $\delta \approx 0.12$, with the oxygen content fixed at 12.00 within experimental uncertainty (Table 1). SEM–EDX gives Ba/Ru = 1.36(2) before thermal cycling and 1.35(5) after cycling, consistent with the refined stoichiometry $4/(3-\delta) = 1.39$ for $\delta \approx 0.12$ (Fig. S3) and indicating no measurable compositional modification upon cycling.

Assuming O = 12.00, the formal average Ru valence is $16/(3-\delta) \approx +5.56$. Discrete Ru^{5+} and Ru^{6+} populations are not assigned in the absence of spectroscopic evidence. Bond-valence-sum (BVS)

analysis for the fully occupied Ru3 site (octahedral; adjacent to, but outside, the face-sharing Ru_3O_{12} trimer units) gives values of 4.21–4.65 at 450 K and 3.98–4.41 at 650 K, depending on the parameter set (B = 0.37 or 0.35; $R_0(Ru^{5+}–O)$ = 1.900 or 1.894 Å; $R_0(Ru^{6+}–O)$ = 1.87 Å).[12–15] The systematically lower BVS values relative to the formal average likely reflect known limitations of BVS under substantial disorder and thermal motion, as well as the use of partial occupancies and displacement parameters in the refinements. The proximity of Ru3 to the disordered trimer network may further broaden the Ru–O bond-length distribution.

S4. Thermal and Chemical Stability

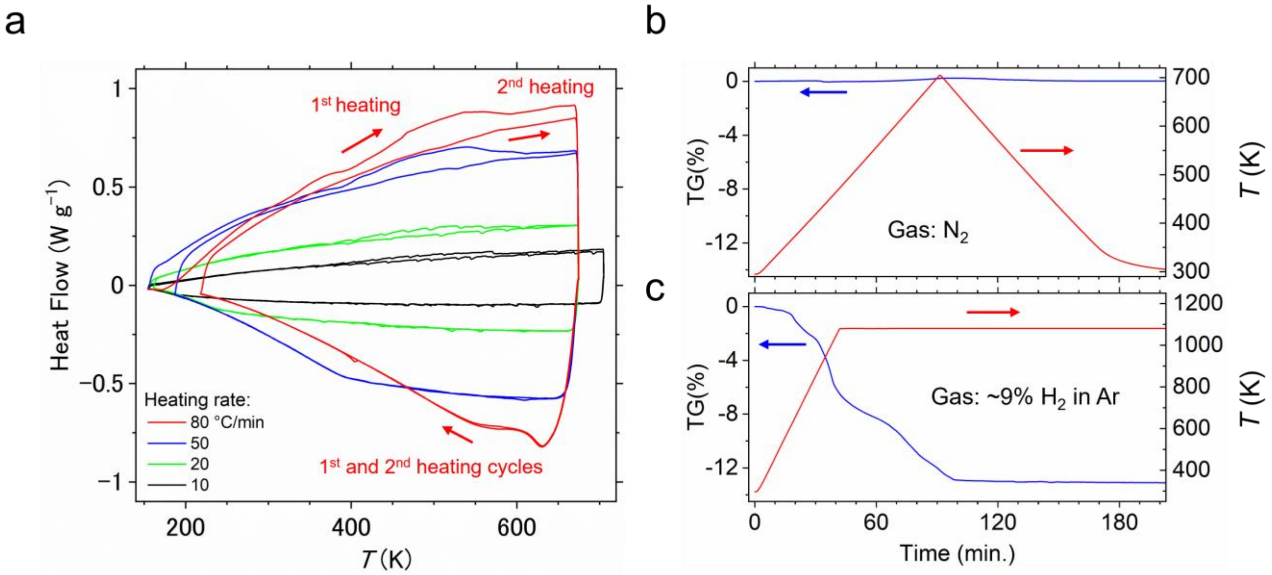

Fig. S4: Thermal analysis of $Ba_4Ru_3O_{12}$.

(a) Differential scanning calorimetry (DSC) curves recorded between 160 and 700 K at heating rates of 10, 20, 50, and 80 K min−1, showing the first and second heating cycles (see arrows). (b) Thermogravimetric (TG) profile under flowing N_2 showing negligible mass change up to 700 K. (c) TG profile under ≈9% H_2 in Ar upon heating to 1073 K. The total mass loss (−12.95%) is slightly larger than expected for $Ba_4Ru_{2.88}O_{12}$ if interpreted solely in terms of oxygen removal. This likely reflects uncertainty associated with volatile Ru-containing species and/or minor gas-stream effects under strongly reducing conditions, while remaining broadly consistent with an oxygen content close to the nominal value under the initial conditions.[16] Spike-like features in (a) were identified as instrumental artifacts based on blank measurements.

S5. Magnetic and Transport Behavior

Magnetic measurements were carried out to examine whether the irreversible structural relaxation is accompanied by changes in magnetic ordering. Field-dependent magnetization curves measured before and after thermal cycling are shown in Fig. S5. The overall magnetic response remains continuous across the temperature range associated with the structural expansion (450–650 K). However, thermal cycling modifies the low-temperature magnetic behavior, indicating that the redistribution process alters the metastable state of the system. Importantly, no abrupt anomaly corresponding to the expansion window is observed, consistent with the absence of an electronic or magnetic phase transition accompanying the structural relaxation.

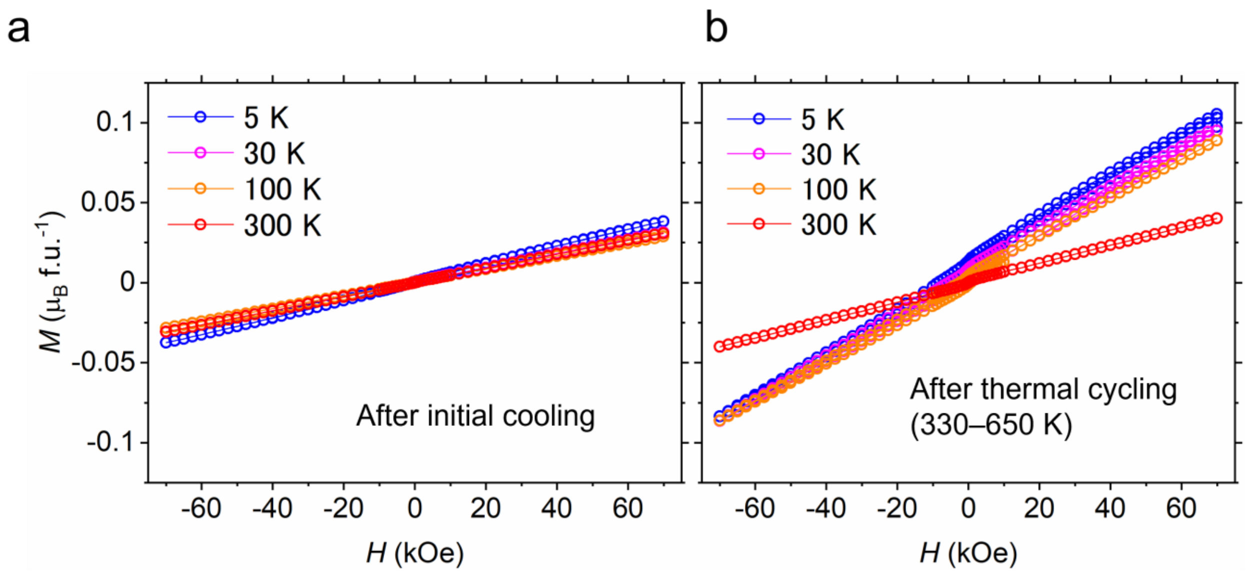

Fig. S5: Magnetization of $Ba_4Ru_3O_{12}$ before and after thermal cycling.

(a) Field-dependent magnetization (*M–H*) measured at 5, 30, 100, and 300 K after initial cooling. (b) *M–H* curves measured at the same temperatures after thermal cycling between 330 and 650 K. Comparison of (a) and (b) shows that thermal cycling alters the low-temperature magnetic response.

S6. Kinetic Analysis from NEB

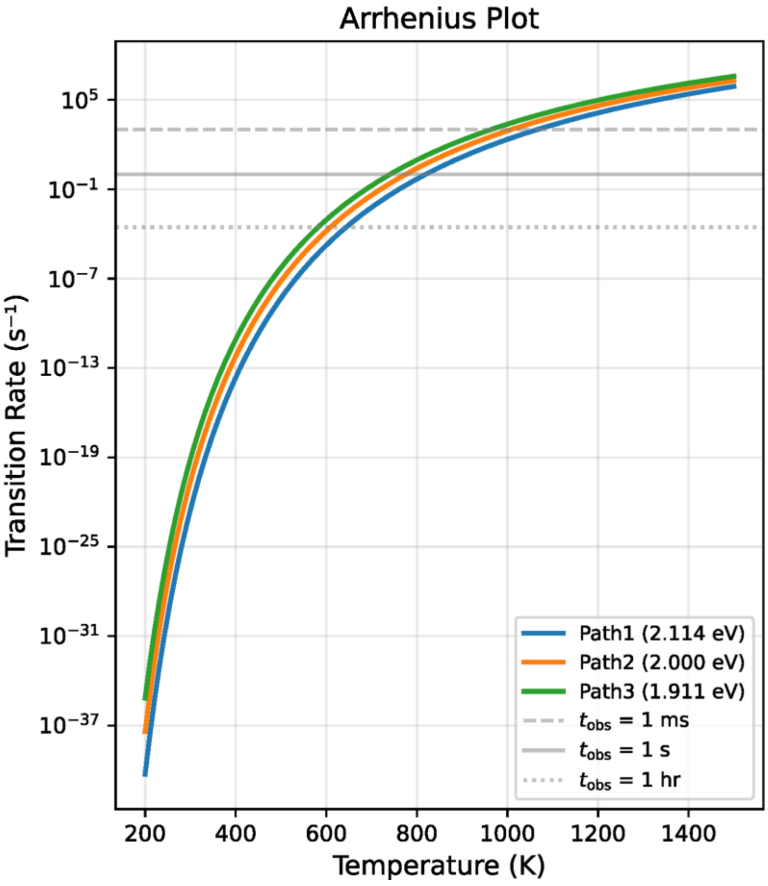

Fig. S6: Arrhenius estimate of Ru-site exchange kinetics from NEB barriers.

Transition rate k as a function of temperature calculated from the Arrhenius relation $k = v_0\exp(-E_a/k_BT)$, using activation energies obtained from nudged elastic band (NEB) calculations for three representative exchange pathways. Path 1, Path 2, and Path 3 correspond to stepwise reductions in R1-site occupancy from 1.00 to 0.833, from 0.833 to 0.666, and from 0.666 to 0.50, respectively, accompanied by corresponding changes in the local Ru arrangements within the Ru_3O_{12} trimers. An attempt frequency $v_0 = 1 \times 10^{13}$ s^{-1} was assumed. Horizontal lines indicate representative observation time scales (1 ms, 1 s, and 1 h), plotted as $k = 1/t_{obs}$. For $E_a \approx 2$ eV, the estimated rates approach the experimentally relevant range within 450–650 K, corresponding to the temperature window of the irreversible expansion.

S7. Benchmark Compilation Methodology

Benchmarking was restricted to bulk, single-phase crystalline materials reported to remain chemically and structurally stable above 400 K, including representative oxides, salts, and alloys. Soft materials (polymers, molecular solids, and halide perovskites) were included for context only and were not used in comparisons restricted to crystalline solids. Thin films, glasses, composites, porous/framework materials, and systems exhibiting melting, decomposition, or irreversible chemical changes within the measurement window were excluded. If a phase transition occurred outside the window, only the single-phase region above 400 K was considered.

For each material, we compiled the maximum volumetric thermal expansion coefficient α_V (K^{-1}) and/or $\Delta V/V$ within the reported stability window. When α_V was not explicitly reported, it was calculated from the temperature derivative of the unit-cell volume. For near-isotropic materials reported only with linear coefficients, $\alpha_V \approx 3\alpha_L$ was used. For anisotropic crystals, α_V was calculated from the full lattice-parameter set when available.

Numerical values were taken from tables or digitized from published figures when necessary. Reported uncertainties were retained as given; when not provided, no additional uncertainty was assigned. The compiled dataset and primary sources are summarized in Fig. S7.

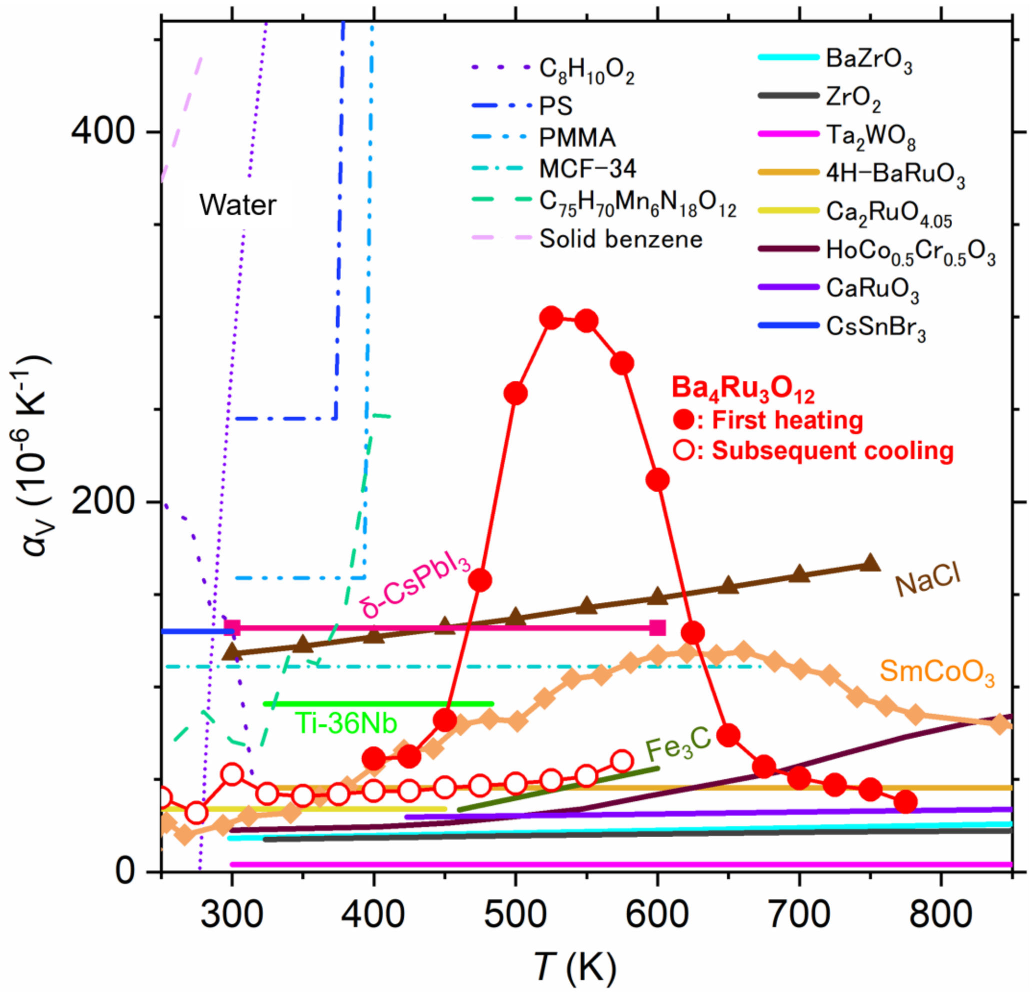

Fig. S7: Extended benchmarking of thermal expansion above 400 K.

Temperature dependence of the volumetric thermal expansion coefficient (α_V) for $Ba_4Ru_3O_{12}$ (red) compared with representative bulk crystalline benchmarks stable above 400 K, including oxides ($SmCoO_3$,[17] $CaRuO_3$,[18] ZrO_2,[19] $BaZrO_3$,[20] 4H–$BaRuO_3$,[21] $Ca_2RuO_{4.05}$,[22] $HoCo_{0.5}Cr_{0.5}O_3$,[23] Ta_2WO_8[24]), salts (NaCl[25]), and alloys (Ti–36Nb,[26] Fe_3C[27]). Non-oxide comparators (δ-$CsPbI_3$,[28] $CsSnBr_3$[29], polystyrene and PMMA,[30,31] water,[32] solid benzene[33]) were included for context only and were not used in comparisons restricted to crystalline oxides. $Ba_4Ru_3O_{12}$ reaches a maximum α_V near 550 K and exceeds the crystalline benchmarks shown within their respective stability windows.

Supplementary References

[1] S. Kawaguchi, M. Takemoto, K. Osaka, E. Nishibori, C. Moriyoshi, Y. Kubota, Y. Kuroiwa, K. Sugimoto, "High-throughput powder diffraction measurement system consisting of multiple MYTHEN detectors at beamline BL02B2 of SPring-8" *Review of Scientific Instruments* **2017**, *88*, 085111.

[2] F. Izumi, K. Momma, "Three-Dimensional Visualization in Powder Diffraction" *Solid State Phenomena* **2007**,

130, 15–20.

[3] K. Momma, F. Izumi, “VESTA: A Three-Dimensional Visualization System for Electronic and Structural Analysis” *Journal of Applied Crystallography* **2008**, *41*, 653–658.

[4] F. Izumi, T. Ikeda, “A Rietveld-Analysis Program RIETAN-98 and its Applications to Zeolites” *Materials Science Forum* **2000**, *321–324*, 198–205.

[5] G. Kresse, J. Furthmüller, “Efficient iterative schemes for ab initio total-energy calculations using a plane-wave basis set” *Physical Review B* **1996**, *54*, 11169–11186.

[6] G. Kresse, J. Hafner, “*Ab initio* molecular dynamics for liquid metals” *Phys. Rev. B* **1993**, *47*, 558–561.

[7] P. E. Blöchl, “Projector augmented-wave method” *Phys. Rev. B* **1994**, *50*, 17953–17979.

[8] J. P. Perdew, K. Burke, M. Ernzerhof, “Generalized Gradient Approximation Made Simple” *Physical Review Letters* **1996**, *77*, 3865–3868.

[9] H. J. Monkhorst, J. D. Pack, “Special points for Brillouin-zone integrations” *Phys. Rev. B* **1976**, *13*, 5188–5192.

[10] G. Henkelman, H. Jónsson, “Improved tangent estimate in the nudged elastic band method for finding minimum energy paths and saddle points” *The Journal of Chemical Physics* **2000**, *113*, 9978–9985.

[11] G. Henkelman, B. P. Uberuaga, H. Jónsson, “A climbing image nudged elastic band method for finding saddle points and minimum energy paths” *The Journal of Chemical Physics* **2000**, *113*, 9901–9904.

[12] I. D. Brown, “What is the best way to determine bond-valence parameters?” *IUCrJ* **2017**, *4*, 514–515.

[13] N. E. Brese, M. O’Keeffe, “Bond-valence parameters for solids” *Acta Crystallographica Section B Structural Science* **1991**, *47*, 192–197.

[14] I. D. Brown, D. Altermatt, “Bond-valence parameters obtained from a systematic analysis of the Inorganic Crystal Structure Database” *Acta Crystallogr B Struct Sci* **1985**, *41*, 244–247.

[15] O. C. Gagné, F. C. Hawthorne, “Comprehensive derivation of bond-valence parameters for ion pairs involving oxygen” *Acta Crystallogr B Struct Sci Cryst Eng Mater* **2015**, *71*, 562–578.

[16] I. Nuta, F. Virot, E. Fischer, C. Chatillon, “Thermodynamic assessment of RuO4 oxide” *Calphad* **2023**, *80*, 102508.

[17] K. Knížek, Z. Jirák, J. Hejtmánek, M. Veverka, M. Maryško, G. Maris, T. T. M. Palstra, “Structural anomalies associated with the electronic and spin transitions in $LnCoO_3$” *Eur. Phys. J. B* **2005**, *47*, 213–220.

[18] R. Ranjan, A. Senyshyn, V. Vashook, R. Niewa, H. Boysen, F. Frey, “Structural stability of conducting oxide CaRuO3 at high temperatures” *Applied Physics Letters* **2007**, *90*, 251913.

[19] R. P. Haggerty, P. Sarin, Z. D. Apostolov, P. E. Driemeyer, W. M. Kriven, “Thermal Expansion of HfO_2 and ZrO_2” *J. Am. Ceram. Soc.* **2014**, *97*, 2213–2222.

[20] Y. Zhao, DonaldJ. Weidner, “Thermal expansion of SrZrO3 and BaZrO3 perovskites” *Phys Chem Minerals* **1991**, *18*, DOI 10.1007/BF00200187.

[21] P. Kayser, S. Injac, B. J. Kennedy, A. L. Menezes De Oliveira, Y. Shirako, M. Hasegawa, “Thermal expansion in $BaRuO_3$ perovskites – an unusual case of bond strengthening at high temperatures” *Dalton Trans.* **2017**, *46*, 2974–2980.

[22] L. Hu, Y. Zhu, Y.-W. Fang, M. Fukuda, T. Nishikubo, Z. Pan, Y. Sakai, S. Kawaguchi, H. Das, A. Machida, T. Watanuki, S. Mori, K. Takenaka, M. Azuma, “Origin and Absence of Giant Negative Thermal Expansion in

Reduced and Oxidized Ca_2RuO_4" *Chem. Mater.* **2021**, *33*, 7665–7674.

[23] V. Hreb, L. Vasylechko, V. Mykhalichko, Y. Prots, "Anomalous Thermal Expansion of $HoCo_{0.5}Cr_{0.5}O_3$ Probed by X-ray Synchrotron Powder Diffraction" *Nanoscale Res Lett* **2017**, *12*, 442.

[24] Y. Luo, Y. Qiao, Q. Gao, J. Wang, J. Guo, X. Ren, M. Chao, Q. Sun, Y. Jia, E. Liang, "Anomalous Thermal Expansion in Ta_2WO_8 Oxide Semiconductor over a Wide Temperature Range" *Inorg. Chem.* **2021**, *60*, 17758–17764.

[25] O. L. Anderson, *Equations of State of Solids for Geophysics and Ceramic Science*, Oxford University Press, New York, NY, **1995**.

[26] M. Bönisch, A. Panigrahi, M. Stoica, M. Calin, E. Ahrens, M. Zehetbauer, W. Skrotzki, J. Eckert, "Giant thermal expansion and α-precipitation pathways in Ti-alloys" *Nat Commun* **2017**, *8*, 1429.

[27] I. G. Wood, L. Vočadlo, K. S. Knight, D. P. Dobson, W. G. Marshall, G. D. Price, J. Brodholt, "Thermal expansion and crystal structure of cementite, Fe_3 C, between 4 and 600 K determined by time-of-flight neutron powder diffraction" *J Appl Crystallogr* **2004**, *37*, 82–90.

[28] A. Marronnier, G. Roma, S. Boyer-Richard, L. Pedesseau, J.-M. Jancu, Y. Bonnassieux, C. Katan, C. C. Stoumpos, M. G. Kanatzidis, J. Even, "Anharmonicity and Disorder in the Black Phases of Cesium Lead Iodide Used for Stable Inorganic Perovskite Solar Cells" *ACS Nano* **2018**, *12*, 3477–3486.

[29] D. H. Fabini, K. Honasoge, A. Cohen, S. Bette, K. M. McCall, C. C. Stoumpos, S. Klenner, M. Zipkat, L. P. Hoang, J. Nuss, R. K. Kremer, M. G. Kanatzidis, O. Yaffe, S. Kaiser, B. V. Lotsch, "Noncollinear Electric Dipoles in a Polar Chiral Phase of $CsSnBr_3$ Perovskite" *J. Am. Chem. Soc.* **2024**, *146*, 15701–15717.

[30] S. Eim, S. Jo, J. Kim, S. Park, D. Lee, T. P. Russell, D. Y. Ryu, "Insights into the Thermal Expansion of Amorphous Polymers" *ACS Macro Lett.* **2024**, *13*, 1490–1494.

[31] R. Ohtani, A. Grosjean, R. Ishikawa, R. Yamamoto, M. Nakamura, J. K. Clegg, S. Hayami, "Zero in-Plane Thermal Expansion in Guest-Tunable 2D Coordination Polymers" *Inorg. Chem.* **2017**, *56*, 6225–6233.

[32] W. Wagner, A. Pruß, "The IAPWS Formulation 1995 for the Thermodynamic Properties of Ordinary Water Substance for General and Scientific Use" *Journal of Physical and Chemical Reference Data* **2002**, *31*, 387–535.

[33] A. Bondi, "Thermal Properties of Molecular Crystals. I. Heat Capacity and Thermal Expansion" *Journal of Applied Physics* **1966**, *37*, 4643–4647.
